# Supplementary material for: The Antifungal Effects of Citral on Magnaporthe oryzae Occur via Modulation of Chitin Content as Revealed by RNA-Seq Analysis
Source: J Fungi (Basel). 2021 Nov 29;7(12):1023. doi: 10.3390/jof7121023 (PMC8704549; doi:10.3390/jof7121023)
Supplement: Supplementary file 1 [file jof-07-01023-s001.zip › Table S1. 8 genes of primer design for RT-qPCR.pdf]

Table S1. 8 genes of primer design for RT-qPCR

Gene	Primer name	Forward primer (5′ −3′)	Reverse primer (5′ −3′)
class III chitinase	*MGG_10333*	ATGTTCGGTCCTCCTCCTGCTG	GGCGGCGAGGTTCTTGATGC
galactose-1-phosphate uridylyltransferase	*MGG_05098*	TCGCAGCCATCGCAACAACTAC	CAATTCCTTGCCTGGTTCCTCTGG
phosphoglucomutase	*MGG_04495*	TTCGCAAGAAGGTCACAGTGTTCC	CGTCCGTCACCGCCAATCAC
glucosamine-6-phosphate isomerase	*MGG_00625*	AATGATAGTGTGCGACGAGGATGC	CCACCTGCTCGATGCTCTTGAAG
FAD binding domain-containing protein	*MGG_01605*	GTCAGCCACCTCTTCACCTTTAGC	TGCCTCGTGTATCCTGGTCTGG
bacteriodes thetaiotaomicron symbiotic chitinase	*MGG_01336*	GGCGGAGGCGAGTTCAACAC	CGGATAAACTGGGCACGGGAAG
bacteriodes thetaiotaomicron symbiotic chitinase	*MGG_03920*	CTGTTCTTCGTCCTGGCGTTCC	AGAAGTAGTGGGTGAGGTCGTAGC
chitin synthase 3	*MGG_09551*	TGTCAAGGCGATAATGGCGGATG	GAACATCAGGAGCGAGGCAACC
