# Supplementary material for: The Antifungal Effects of Citral on Magnaporthe oryzae Occur via Modulation of Chitin Content as Revealed by RNA-Seq Analysis
Source: J Fungi (Basel). 2021 Nov 29;7(12):1023. doi: 10.3390/jof7121023 (PMC8704549; doi:10.3390/jof7121023)
Supplement: Supplementary file 1 [file jof-07-01023-s001.zip › Table S2. Summary of reference genetic data from processed samples..pdf]

Table S2. Summary of reference genetic data from processed samples.

Sample	Valid Data Read	Valid Data Read	Valid Ratio(reads)	Valid_Base (Gb)	Q20%	Q30%	GC content%
a0_1	40190860	40190860	82.91	6.03G	99.99	99.29	53.00
a0_2	32637092	32637092	81.62	4.90G	99.98	99.26	52.00
a0_3	31574984	31574984	79.46	4.74G	99.99	99.27	52.00
A2_1	44460028	44460028	84.06	6.67G	99.98	99.11	51.50
A2_2	41826300	41826300	85.03	6.27G	99.98	99.23	52.00
A2_3	38927102	38927102	78.64	5.84G	99.98	99.19	51.00
A3_1	35677366	35677366	82.45	5.35G	99.98	99.08	51.00
A3_2	32999572	32999572	79.27	4.95G	99.98	99.18	51.50
A3_3	38102392	38102392	82.12	5.72G	99.98	99.11	52.00
A4_1	35256260	35256260	85.73	5.29G	99.98	99.16	52.00
A4_2	35937312	35937312	86.01	5.39G	99.98	99.22	52.00
A4_3	42175516	42175516	88.76	6.33G	99.98	99.23	52.00
b0_1	40516006	40516006	81.71	6.08G	99.98	99.22	52.00
b0_2	41085158	41085158	87.09	6.16G	99.98	99.26	53.00
b0_3	39467774	39467774	84.46	5.92G	99.98	99.24	52.50
B2_1	36655378	36655378	79.69	5.50G	99.98	99.10	52.00
B2_2	31181238	31181238	75.23	4.68G	99.98	99.19	52.00
B2_3	34309364	34309364	77.08	5.15G	99.98	99.11	52.00
B3_1	38055198	38055198	82.14	5.71G	99.98	99.16	52.00
B3_2	35762606	35762606	81.61	5.36G	99.98	99.22	52.00
B3_3	40124154	40124154	83.27	6.02G	99.98	99.23	52.00
B4_1	37941296	37941296	87.92	5.69G	99.98	99.17	52.00
B4_2	33574620	33574620	83.61	5.04G	99.98	99.17	52.00
B4_3	34245100	34245100	81.71	5.14G	99.98	99.22	52.00
c0_1	39991880	39991880	85.76	6.00G	99.98	99.24	52.00
c0_2	37878658	37878658	84.75	5.68G	99.98	99.20	52.00
c0_3	36572658	36572658	86.05	5.49G	99.98	99.36	53.00
C2_1	37354334	37354334	79.16	5.60G	99.98	99.12	52.00
C2_2	32418936	32418936	85.09	4.86G	99.98	99.26	53.00
C2_3	32196282	32196282	76.23	4.83G	99.98	99.18	52.00
C3_1	31206078	31206078	77.84	4.68G	99.98	99.10	52.00
C3_2	33382398	33382398	75.63	5.01G	99.98	99.09	51.50
C3_3	36485950	36485950	80.62	5.47G	99.98	99.11	52.00
C4_1	37824604	37824604	87.01	5.67G	99.98	99.25	53.00
C4_2	44578076	44578076	88.48	6.69G	99.99	99.26	53.00
C4_3	37807386	37807386	88.17	5.67G	99.99	99.26	52.00

a0, A2, A3, A4.*Magnaporthe oryzae* treating with 0 µg/mL citral, b0, B1, B2, B3, B4. *M. oryzae* treating with 50 µg/mL , c0, C1, C2, C3, C4 *M. oryzae* treating with 100 µg/mL. There are three repetitions for each treatment.
