# Supplementary material for: The Antifungal Effects of Citral on Magnaporthe oryzae Occur via Modulation of Chitin Content as Revealed by RNA-Seq Analysis
Source: J Fungi (Basel). 2021 Nov 29;7(12):1023. doi: 10.3390/jof7121023 (PMC8704549; doi:10.3390/jof7121023)
Supplement: Supplementary file 1 [file jof-07-01023-s001.zip › Table S3. Comparison of transcriptome sequencing and reference genome of M. oryzae under citral stress.pdf]

Table S3. Comparison of transcriptome sequencing and reference genome of *M. oryzae* under citral stress.

Sample	Valid reads	Mapped reads	Unique Mapped reads	exon	intron	intergenic
a0_1	40190860	37155714(92.45%)	34573529(86.02%)	98.41	1.42	0.17
a0_2	32637092	30329975(92.93%)	28206299(86.42%)	98.12	1.66	0.22
a0_3	31574984	29336859(92.91%)	27058394(85.70%)	98.49	1.34	0.17
A2_1	44460028	40219396(90.46%)	37268729(83.83%)	98.36	1.45	0.19
A2_2	41826300	39277917(93.91%)	36353023(86.91%)	98.40	1.44	0.17
A2_3	38927102	35982339(92.44%)	33521134(86.11%)	98.17	1.62	0.21
A3_1	35677366	31937455(89.52%)	29487380(82.65%)	98.22	1.59	0.19
A3_2	32999572	30016444(90.96%)	27860653(84.43%)	98.23	1.58	0.18
A3_3	38102392	35325305(92.71%)	32635942(85.65%)	98.22	1.60	0.18
A4_1	35256260	32652750(92.62%)	30084677(85.33%)	97.84	1.98	0.18
A4_2	35937312	32898493(91.54%)	30657382(85.31%)	98.03	1.79	0.18
A4_3	42175516	39411488(93.45%)	36599078(86.78%)	97.81	2.01	0.18
b0_1	40516006	36972479(91.25%)	34291973(84.64%)	98.49	1.27	0.24
b0_2	41085158	38577768(93.90%)	35654650(86.78%)	98.42	1.35	0.24
b0_3	39467774	36538317(92.58%)	33783039(85.60%)	98.51	1.25	0.24
B2_1	36655378	33037151(90.13%)	30357455(82.82%)	98.23	1.56	0.21
B2_2	31181238	28674010(91.96%)	26512300(85.03%)	98.15	1.65	0.21
B2_3	34309364	31295736(91.22%)	28816637(83.99%)	98.25	1.54	0.22
B3_1	38055198	34613739(90.96%)	31963455(83.99%)	98.04	1.77	0.20
B3_2	35762606	32688873(91.41%)	30357527(84.89%)	98.09	1.72	0.19
B3_3	40124154	37251610(92.84%)	34480202(85.93%)	98.08	1.73	0.20
B4_1	37941296	34980924(92.20%)	32294796(85.12%)	98.29	1.51	0.20
B4_2	33574620	30436056(90.65%)	28126387(83.77%)	98.31	1.50	0.19
B4_3	34245100	31522775(92.05%)	29330914(85.65%)	98.34	1.47	0.20
c0_1	39991880	36855518(92.16%)	34205704(85.53%)	98.44	1.36	0.20
c0_2	37878658	35613103(94.02%)	33013316(87.16%)	98.39	1.43	0.18
c0_3	36572658	33754659(92.29%)	31530601(86.21%)	98.71	1.11	0.17
C2_1	37354334	33073204(88.54%)	30510572(81.68%)	98.43	1.36	0.21
C2_2	32418936	29334009(90.48%)	27162114(83.78%)	98.67	1.16	0.17
C2_3	32196282	29459254(91.50%)	27352757(84.96%)	98.55	1.25	0.20
C3_1	31206078	29001333(92.93%)	26638596(85.36%)	98.72	1.10	0.18
C3_2	33382398	30176492(90.40%)	27946201(83.72%)	98.71	1.10	0.19
C3_3	36485950	33910068(92.94%)	31394261(86.04%)	98.68	1.12	0.19
C4_1	37824604	35026515(92.60%)	32454235(85.80%)	98.44	1.39	0.16
C4_2	44578076	41131557(92.27%)	38230696(85.76%)	98.47	1.37	0.17
C4_3	37807386	35035661(92.67%)	32615798(86.27%)	98.45	1.39	0.16

a0, A2, A3, A4. *M. oryzae* treating with 0 μg/mL citral, b0, B1, B2, B3, B4. *M. oryzae* treating with 50 μg/mL , c0, C1, C2, C3, C4 *M. oryzae* treating with 100 μg/mL. There are three repetitions for each treatment.
