# Supplementary material for: The Antifungal Effects of Citral on Magnaporthe oryzae Occur via Modulation of Chitin Content as Revealed by RNA-Seq Analysis
Source: J Fungi (Basel). 2021 Nov 29;7(12):1023. doi: 10.3390/jof7121023 (PMC8704549; doi:10.3390/jof7121023)
Supplement: Supplementary file 1 [file jof-07-01023-s001.zip › Table S4. Large value distribution statistics of reference gene table for transcriptome sequencing of M. oryzae under citral stress.pdf]

Table S4. Large value distribution statistics of reference gene table for transcriptome sequencing of *M. oryzae* under citral stress.

Sample	Exp gene	Min.	1st Qu.	Median	Mean	3rd Qu.	Max.	Sd.	Sum.
a0_1	13053	0.00	0.42	6.28	51.50	25.89	13666.67	284.15	672283.16
a0_2	13053	0.00	0.14	4.38	47.60	20.40	22744.68	317.37	621285.25
a0_3	13053	0.00	0.16	6.08	50.71	25.56	10496.43	258.63	661892.17
b0_1	13053	0.00	0.04	3.40	50.88	17.57	17797.61	301.25	664168.39
b0_2	13053	0.00	0.08	3.41	50.48	17.98	18036.74	297.36	658867.19
b0_3	13053	0.00	0.10	3.45	50.90	17.86	18652.30	303.48	664339.56
c0_1	13053	0.00	0.00	1.94	49.95	11.05	29743.22	476.18	651986.49
c0_2	13053	0.00	0.00	1.83	48.65	10.57	25141.15	451.48	635078.48
c0_3	13053	0.00	0.12	2.70	51.91	13.90	22815.19	424.20	677623.87
A2_1	13053	0.00	0.40	7.47	49.40	28.32	18671.75	267.81	644815.79
A2_2	13053	0.00	0.33	6.02	50.14	25.06	10643.17	253.70	654449.07
A2_3	13053	0.00	0.22	5.95	48.60	24.31	22793.38	305.46	634404.27
B2_1	13053	0.00	0.06	6.67	49.97	25.41	14181.24	265.00	652271.56
B2_2	13053	0.00	0.03	6.83	49.96	25.75	14496.08	267.19	652096.71
B2_3	13053	0.00	0.06	6.67	50.27	25.25	13953.27	267.05	656121.53
C2_1	13053	0.00	0.45	7.79	47.64	30.61	12766.48	224.52	621820.35
C2_2	13053	0.00	0.46	7.70	51.70	28.09	36563.15	380.14	674845.72
C2_3	13053	0.00	0.35	7.71	47.36	30.82	11651.12	213.76	618165.99
A3_1	13053	0.00	0.32	6.53	48.13	26.13	22481.96	282.62	628203.33
A3_2	13053	0.00	0.23	6.61	48.21	25.79	22812.56	284.89	629220.69
A3_3	13053	0.00	0.09	6.37	46.71	25.69	22139.84	275.78	609764.24
B3_1	13053	0.00	0.45	7.16	49.47	26.94	12571.48	253.60	645675.35
B3_2	13053	0.00	0.37	7.24	49.90	26.50	12564.43	258.47	651408.03
B3_3	13053	0.00	0.38	7.15	49.46	26.97	11786.47	250.96	645613.67
C3_1	13053	0.00	0.21	6.33	52.04	24.35	17917.82	298.37	679311.21
C3_2	13053	0.00	0.00	6.10	52.33	23.77	18352.98	306.86	683097.37
C3_3	13053	0.00	0.15	6.18	52.14	24.38	18264.81	304.97	680630.26
A4_1	13053	0.00	0.36	5.44	46.46	22.55	17490.75	259.44	606467.31
A4_2	13053	0.00	0.53	6.94	46.82	26.87	20228.89	264.64	611109.71
A4_3	13053	0.00	0.42	5.39	46.36	22.75	17531.29	259.77	605102.69
B4_1	13053	0.00	0.63	7.94	50.26	28.37	15808.50	269.41	656095.16
B4_2	13053	0.00	0.49	7.87	50.33	28.12	16329.18	272.65	656950.52
B4_3	13053	0.00	0.51	7.82	50.46	27.97	16214.79	273.26	658662.60
C4_1	13053	0.00	0.54	7.67	51.92	27.50	15139.83	287.17	677759.28
C4_2	13053	0.00	0.54	7.73	51.94	27.36	14999.00	290.87	677972.89
C4_3	13053	0.00	0.58	7.73	52.04	27.14	15040.03	292.16	679289.80

a0, A2, A3, A4. *M. oryzae* treating with 0 μg/mL citral, b0, B1, B2, B3, B4. *M. oryzae* treating with 50 μg/mL , c0, C1, C2, C3, C4 *M. oryzae* treating with 100 μg/mL. There are three repetitions for each treatment.
