# Supplementary figures and images for: The Antifungal Effects of Citral on Magnaporthe oryzae Occur via Modulation of Chitin Content as Revealed by RNA-Seq Analysis
Source: J Fungi (Basel). 2021 Nov 29;7(12):1023. doi: 10.3390/jof7121023 (PMC8704549; doi:10.3390/jof7121023)

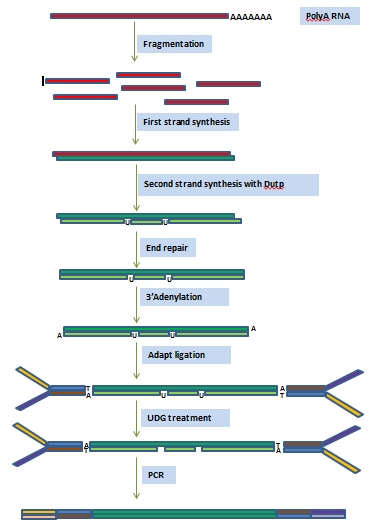

Supplement: Supplementary file 1 [file jof-07-01023-s001.zip › Figure S1. Experimental flow.png]

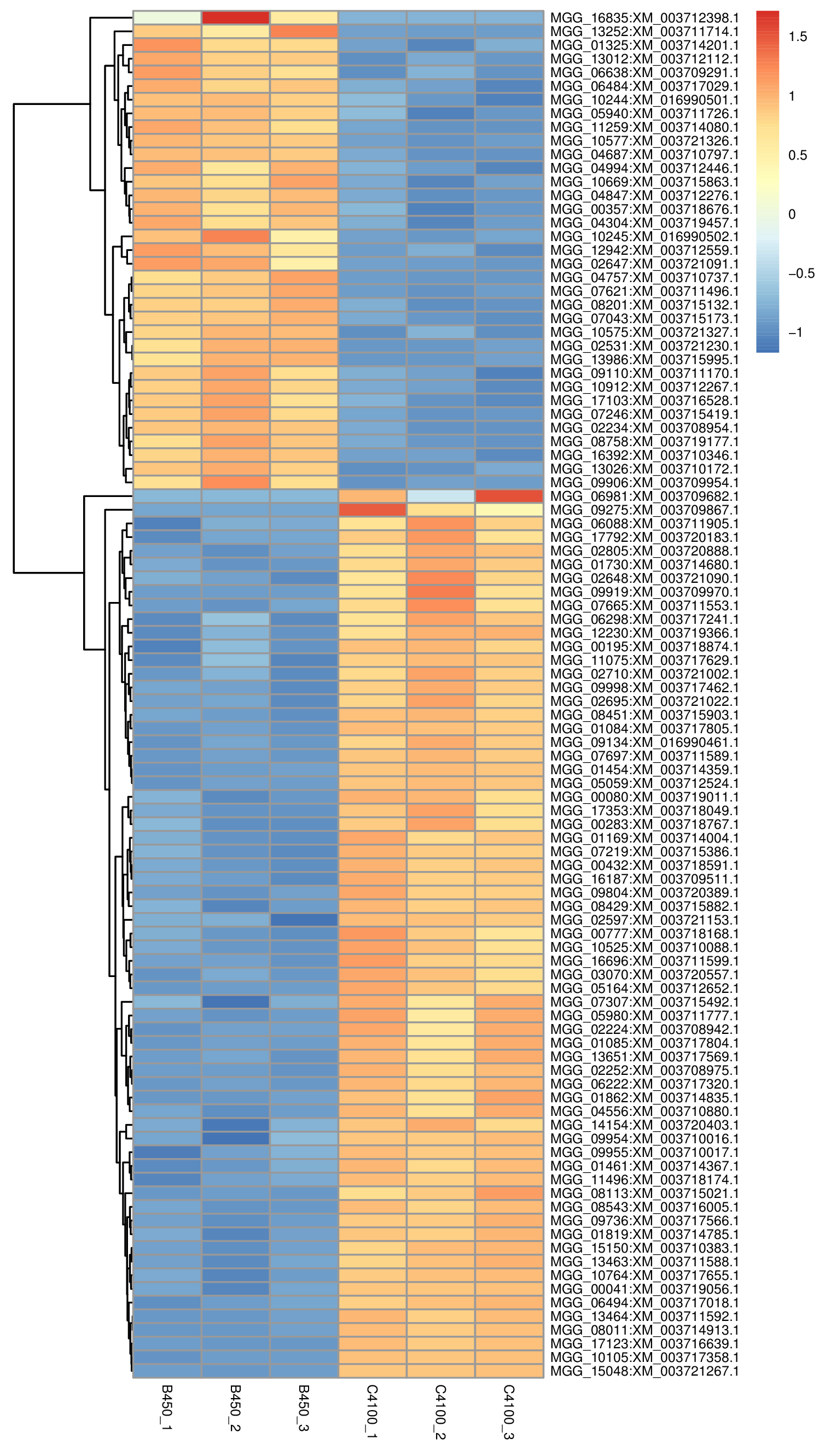

Supplement: Supplementary file 1 [file jof-07-01023-s001.zip › Figure S2. Systematic clustering of differentially expressed genes (DEG) in 36 transcriptome libraries.png]

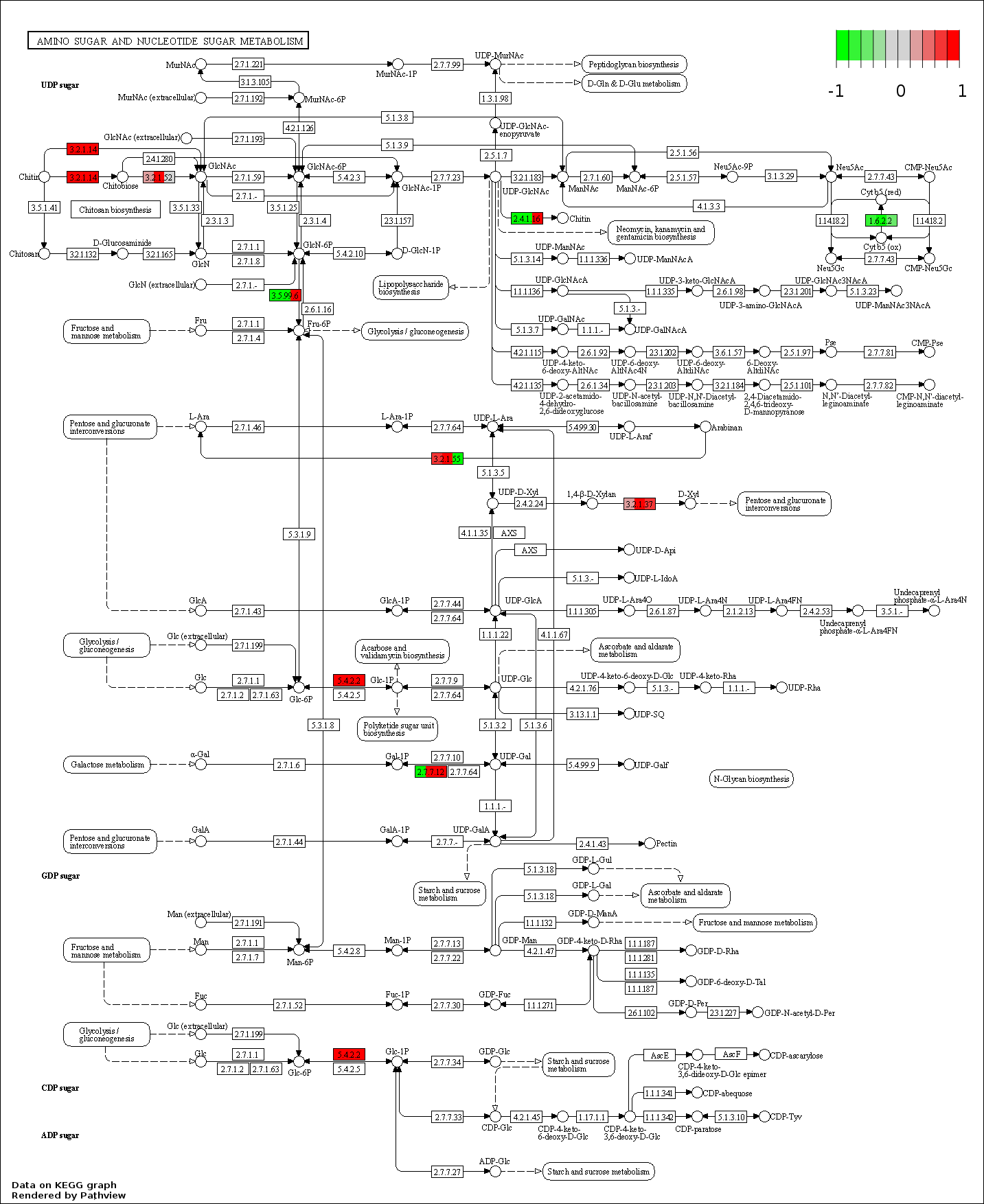

Supplement: Supplementary file 1 [file jof-07-01023-s001.zip › Figure S3. Pathway of amino and nucleotide sa╠gar metabolism of M. oryzae.png]
